# A network pharmacology- and transcriptomics-based investigation reveals an inhibitory role of β-sitosterol in glioma via the EGFR/MAPK signaling pathway: Inhibitory role of β-sitosterol in glioma via EGFR/MAPK pathway

**DOI:** 10.3724/abbs.2023251

**Published:** 2023-12-22

**Authors:** Yufang Xie, Zhijian Chen, Shuang Li, Meijuan Yan, Wenjun He, Li Li, Junqiang Si, Yan Wang, Xinzhi Li, Ketao Ma

**Affiliations:** 1 Key Laboratory of Xinjiang Endemic and Ethnic Diseases Ministry of Education Shihezi University School of Medicine Shihezi 832000 China; 2NHC Key Laboratory of Prevention and Treatment of Central Asia High Incidence Diseases First Affiliated Hospital Shihezi University School of Medicine Shihezi 832000 China; 3 Department of Physiology Shihezi University School of Medicine Shihezi 832000 China; 4 Department of Pathophysiology Shihezi University School of Medicine Shihezi 832000 China

**Keywords:** β-sitosterol, glioma, proliferation, G2/M phase arrest, apoptosis, EGFR/MAPK signaling pathway

## Abstract

Glioma is characterized by rapid cell proliferation, aggressive invasion, altered apoptosis and a poor prognosis. β-Sitosterol, a kind of phytosterol, has been shown to possess anticancer activities. Our current study aims to investigate the effects of β-sitosterol on gliomas and reveal the underlying mechanisms. Our results show that β-sitosterol effectively inhibits the growth of U87 cells by inhibiting proliferation and inducing G2/M phase arrest and apoptosis. In addition, β-sitosterol inhibits migration by downregulating markers of epithelial-mesenchymal transition (EMT). Mechanistically, network pharmacology and transcriptomics approaches illustrate that the EGFR/MAPK signaling pathway may be responsible for the inhibitory effect of β-sitosterol on glioma. Afterward, the results show that β-sitosterol effectively suppresses the EGFR/MAPK signaling pathway. Moreover, β-sitosterol significantly inhibits tumor growth in a U87 xenograft nude mouse model. β-Sitosterol inhibits U87 cell proliferation and migration and induces apoptosis and cell cycle arrest in U87 cells by blocking the EGFR/MAPK signaling pathway. These results suggest that β-sitosterol may be a promising therapeutic agent for the treatment of glioma.

## Introduction

Gliomas include well-differentiated low-grade astrocytomas, oligodendrogliomas, and glioblastoma multiforme (GBM) [1]. GBM is the most common primary malignant tumor of the central nervous system, with an incidence of approximately 5–8 cases per 100,000 people per year [ 2, 3]. GBM is characterized by abnormal angiogenesis, high invasiveness and altered apoptosis [4]. The standard therapy for glioma is surgical resection of the tumor, followed by concomitant temozolomide chemotherapy and adjuvant radiotherapy [5]. However, current anticancer drugs may cause various adverse effects, with a patient mean survival of only 15 months [6]. Therefore, there is an urgent need to develop new drugs to treat this deadly disease.

Natural antioxidants and many phytochemicals are recommended as anticancer adjuvant therapies because of their obvious antitumour activity [ 7, 8]. Currently, approximately 100 compounds derived from natural products and their modification products, such as paclitaxel, camptothecin, curcumin, and teniposide, are approved due to their anticancer activities [ 9, 10]. Thus, searching for anticancer agents/compounds from plants has become a significant way to develop anticancer drugs [ 11, 12]. Phytosterols are widely distributed in herbs, fungi and animals [13]. β-Sitosterol, a kind of phytosterol, is a major component of the human diet and has many functions in promoting health and mitigating disease [14]. β-Sitosterol plays an important role in the prevention and treatment of cancers. It can inhibit proliferation and induce apoptosis in cancer cells [15]. Recent studies have shown that β-sitosterol can exhibit anti-pancreatic cancer activity by modulating apoptosis and inhibiting epithelial-mesenchymal transition (EMT) by deactivating Akt/GSK-3β signaling [16]. β-Sitosterol exerts anticancer effects on AGS cells and xenograft mouse models by mediating AMPK, PTEN, and Hsp90 [17]. Furthermore, β-sitosterol can significantly inhibit the growth of A549 cells and trigger apoptosis via ROS-mediated mitochondrial dysregulation [18]. However, there is no literature that mentions the role of β-sitosterol in glioma, and the specific mechanisms of the effects remain unclear. This work was designed to assess the potential impact of β-sitosterol on glioma.

With the development of systemic biological research, network pharmacology is a more appropriate approach to identify drug-gene-disease links, explaining the complicated mechanism of drugs at the molecular level [ 19, 20]. Transcriptome analysis based on high-throughput sequencing provides a rapid method to identify mRNA changes after treatment and helps to identify the underlying mechanisms of traditional Chinese medicine (TCM) [ 21, 22]. Recent studies have shown that high-throughput RNA sequencing (RNA-seq) has been used for pathogenesis research and biomarker screening in the diagnosis and prognosis of complex diseases such as cancer, diabetes and neurodegenerative diseases [23]. Importantly, it can provide a basis for effectively validating the predicted results of network pharmacology.

In this study, we conducted *in vitro* and *in vivo* experiments to evaluate the effect of β-sitosterol on U87 cells. Subsequently, network pharmacology and transcriptomics methods were used to further study the underlying mechanism of β-sitosterol’s effects. Finally, molecular biology experiments were used to verify the mechanism of antiglioma.

## Materials and Methods

### Materials and cell culture

β-Sitosterol (molecular formula: C _29_H _500_; batch molecular weight: 414.69; purity: >98%; Cat No. 83-46-5) was purchased from Yuanye Biotechnology (Shanghai, China). β-Sitosterol was dissolved in dimethyl sulfoxide (DMSO) to 5 g/L and diluted in fresh medium to the desired concentration. The final concentration of DMSO in the fresh medium did not exceed 0.1%, and DMSO at this concentration had no toxic effect on cells.

The glioma cell line U87 was purchased from the Stem Cell Bank, Chinese Academy of Sciences (Shanghai, China). The cell line was cultured in DMEM (Gibco, Carlsbad, USA) supplemented with 10% fetal bovine serum (Biological Industries, Kibbutz Beit Haemek, Israel), 100 U/mL penicillin and 100 μg/mL streptomycin (HyClone, Logan, USA). The cell line was maintained at 37 °C with 5% CO _2_.

### MTT assay

The effects of β-sitosterol on cell proliferation and viability were measured by the MTT assay [24]. In brief, U87 cells (5×10 ^4^ cells/mL) were washed with fresh media and seeded in 96-well plates with 100 μL of medium. After the cells adhered to the wall, they were incubated with β-sitosterol (0, 10, 20, 30, 40, and 50 μM) for 24, 48, or 72 h. After incubation with different concentrations of β-sitosterol for various time points, fresh medium containing 10 μL of MTT (5 mg/mL) was added to each well for 4 h. Then, 100 μL of DMSO was added to each well and shaken for 10 min in the dark. The absorbance at 570 nm was measured using a microplate reader (BioTek Instruments, Winooski, USA). Cell viability is shown as percent cell viability compared with the control group.

### Colony formation assay

U87 cells were trypsinized and seeded in 6-well plates at a density of 1000 cells per well. After incubation for 24 h, U87 cells were treated with different concentrations of β-sitosterol and cultured for 48 h. Next, the culture medium containing the drug was discarded and replaced by drug-free complete medium. The treated cells were cultured for 15 days to allow colony formation. Cells were fixed with paraformaldehyde (HyClone) for 20 min at room temperature (24‒26°C) and stained with 1% crystal violet solution for 15 min. Colonies were inspected and photographed using an inverted microscope (Olympus, Tokyo, Japan). Colonies of more than 50 cells were counted to calculate the colony formation rate.

### Morphological assay and Hoechst 33342 staining

U87 cells were plated into 6-well plates and cultured for 24 h. After incubation with the indicated concentrations of β-sitosterol for 48 h, the cells were washed with PBS and fixed in 4% paraformaldehyde for 20 min at room temperature (24‒26°C). Next, the cells were stained with 10 μg/mL Hoechst 33342 (Solarbio, Beijing, China) for 30 min in the dark at 37°C. The cells were washed with cold PBS, and morphological changes and nuclear condensation in the cells were observed and photographed at 100× magnification using a fluorescence microscope (Olympus).

### Cell cycle analysis

Cells were seeded in a 6-well culture plate and treated with different concentrations of β-sitosterol for 48 h. After treatment, the cells were collected by digestion, and cell suspensions were prepared. The cells were fixed in 70% precooled ethanol solution overnight at 4°C and then centrifuged at 800 *g* for 5 min. The ethanol solution was decanted, and the cells were resuspended in PBS and washed twice. Then, they were resuspended in 500 μL of PBS containing 40 μg/mL propidium iodide (PI) (MultiSciences Lianke Biotech Co., Ltd., Hangzhou, China) and 1 mL of DNA staining solution for 30 min in the dark at room temperature. The content of PI-DNA in each sample was detected with a BD FACSAria III flow cytometer (BD Bioscience, Bedford, USA) and analyzed using the FlowJo 10.6.2 software (BD Bioscience).

### Apoptosis assay

β-Sitosterol-induced apoptosis in U87 cells was detected using an Annexin-V-FITC staining kit (MultiSciences Lianke Biotech Co., Ltd.) according to the manufacturer’s instructions. U87 cells were seeded on a 6-well culture plate and treated with different concentrations of β-sitosterol for 48 h. The treated cells were trypsinized and made into cell suspensions in 500 μL of 1× binding buffer containing 5 μL of Annexin V-FITC and 10 μL of PI for 5 min in the dark. After the reaction, the apoptotic rates were analyzed using a BD FACSAria III flow cytometer.

### Wound healing assay

Logarithmic growth phase cells were collected and seeded at a density of 1×10 ^5^ cells/mL in 6-well plates. When the cells grew to 90% confluence, the monolayer was scratched using a pipette tip, and the exfoliated cells were gently washed with serum-free medium. Next, the cells were treated with different concentrations of β-sitosterol to detect cell migration at 0 h and 48 h. The level of migration was photographed under an inverted microscope (Olympus). Image-Pro Plus software was used to measure the area before and after the scratch and calculate the migration area.

### Transwell assay

U87 cells (1×10 ^5^ cells/well) were suspended in low serum (2% FBS) medium and seeded into the upper chamber of a 24-micron Transwell plate (Corning, New York, USA). The cells were treated with various concentrations of β-sitosterol for 48 h in the upper chamber. Then, the lower chamber was filled with fresh complete medium containing 10% FBS. After incubation for 48 h, the cells attached to the upper surface of the filter membranes were washed and fixed, and the migrated cells were stained with 1% crystal violet. The level of migration was observed using an inverted microscope (Olympus).

### Prediction of the potential targets of β-sitosterol

Information on β-sitosterol was obtained from the literature and TCMSP ( https://tcmspw.com/tcmsp.php). Oral bioavailability (OB)≥30%, drug similarity (DL)≥0.18 and blood-brain barrier (BBB)≥‒0.3 were assigned as the criteria for screening active constituents [6]. The chemical structure of β-sitosterol was retrieved from PubChem ( https://pubchem.ncbi.nlm.nih.gov/), and putative targets were predicted from the target prediction database Swiss Target Prediction ( www.swisstargetprediction.ch). The target name was uploaded to the UniProt database ( https://www.uniprot.org/), and *Homo sapiens* was selected for the species. Then, the targets associated with β-sitosterol were obtained.

### Potential target genes for glioma

For disease target identification, the keywords used in the search were limited to ″glioma″. The DisGeNET ( https://www.disgenet.org/), OMIM ( https://www.omim.org/) and GeneCards databases ( https://www.genecards.org/) were used to retrieve glioma cancer-related genes [25]. The target genes retrieved from the two databases were merged, and duplicate items were eliminated to acquire all target genes of glioma. The Venn diagram web tool ( http://bioinformatics.psb.ugent.be/webtools/Venn/) was applied to draw the Venn map of the potential targets of β-sitosterol and the disease targets of glioma.

### Network construction and analysis

The STRING database provides different protein-protein correlation interactions and their interactive levels based on confidence scoring. The β-sitosterol-regulated glioma targets were entered into STRING ( https://string-db.org/) to obtain relevant information on protein-protein interactions (PPIs). The potential targets were imported into Cytoscape v3.6.1 software to visualize the PPI network and obtain the core targets with degree values higher than the average node.

### Functional enrichment analysis

The omicshare platform ( https://www.omicshare.com/) was used to carry out GO enrichment analysis and KEGG pathway annotation [26]. The results were sequenced by using an adjusted *P* value <0.05, and the top 20 GO enrichment terms and KEGG pathways with higher counts were analyzed.

### Molecular docking

The crystal structures of key antiglioma targets in β-sitosterol were obtained from the RCSB Protein Data Bank ( https://www.pdb.org/),and the structure of β-sitosterol was downloaded from PubChem. PyMol (version 2.5.0) was used to process proteins, including removing the ligands, correcting the protein structure, and removing water. AutoDockTools 1.5.6 software was used for docking programs, and PyMOL software was applied for the final visualization. In addition, Schrodinger’s Maestro 12.8 modelling suite was employed to obtain 2D structures [27].

### RNA sequencing

After treatment with different concentrations of β-sitosterol for 48 h, U87 cells were collected and washed with PBS twice. A total of 8 samples were available, including 4 samples from the β-sitosterol group and 4 samples from the control group. Total RNA was isolated using a PureLink RNA mini kit (Thermo Fisher Scientific, Waltham, USA) following the manufacturer’s instructions. Library construction and mRNA sequencing were conducted by NovelBio (Shanghai, China). mRNA was enriched from total RNA by oligo (dT) beads. The enriched mRNA was fragmented and reverse transcribed into cDNA. The purified cDNA was sequenced with paired-end 150 bp reads on an Illumina HiSeq X platform. The β-sitosterol and control groups were analyzed by the DESeq2 R package (1.16.1) to determine the differentially expressed genes (DEGs). *P* value <0.05 and fold change ≥1.5 were set as the thresholds for statistical significance. We carried out KEGG pathway and GO enrichment analysis annotation for predicting the targets of β-sitosterol in glioma by the R software package Bioconductor (version 3.6.1). In our study, the top 20 GO enrichment terms or KEGG pathways were presented and analyzed by clusterProfiler [28].

### Detection of relative expression levels of *SOS1* and *EGFR* by RT-PCR

β-Sitosterol interfered with U87 cells, total RNA was extracted using Trizol (Invitrogen, Carlsbad, USA), mRNA concentration and purity were determined, complementary DNA (cDNA) was synthesized according to the instructions of the reverse transcription kit (TaKaRa, Dalian, China), and amplification was performed according to the instructions of the fluorescence quantitative RT-PCR kit (TaKaRa). Reaction conditions were as follows: 95°C for 30 s, 95°C for 5 s, 60°C for 30 s, cycling 40 times. Using *β-actin* as an internal reference, the relative expression levels of *SOS1* and *EGFR* mRNA were calculated by the 2 ^–△△Ct^ method. The list of primers used in this study is shown in Table 1.

**Table:** **Table 1** Sequences of primers used for real-time PCR

Gene	Sequence (5′→3′)
*SOS1*	Forward	TGCAGCTAGGGATGTGAATCTTC
Reverse	GGAGCCCAGTCCATCAGAACT
*EGFR*	Forward	AACACAGTGGAGCGAATTCCTTT
Reverse	GGAAGTCCATCGACATGTTGCT
*β-actin*	Forward	TGTGATGGTGGGAATGGGTCAG
Reverse	TTTGATGTCACGCACGATTTCC

### Western blot analysis

U87 cells were seeded at a density of 1×10 ^5^ cells/mL on a 60 mm plate and treated with β-sitosterol at the indicated concentrations for 48 h. The total proteins were quantified by using a BCA protein assay kit (Biosharp, Shanghai, China).

Then, equal amounts of total protein were separated by SDS-PAGE and transferred onto PVDF membranes (Millipore, Billerica, USA). After blocking in 5% bovine serum albumin (BSA) solution for 2 h, specific primary antibodies were incubated with the membranes overnight at 4°C. The incubated membranes were washed with TBST. Next, secondary antibodies were incubated with the washed membranes for 2 h. After washing, the blots were developed with enhanced chemiluminescence (Thermo Fisher Scientific) and visualized on a Tanon 5200 imaging analysis system (Tanon, Beijing, China). The antibodies used are shown in Table 2.

**Table:** **Table 2** Antibodies used for western blot analysis

Name	Dilution	Company
Bax	1:2000	Abcam, Cambridge, UK
Bcl-2	1:2000	Abcam
CDK-1	1:1000	Abcam
Cleaved caspase-3	1:1000	Abcam
Cycb1	1:1000	Abcam
PCNA	1:1000	Boster, Wuhan, China
E-Cadherin	1:1000	CST, Boston, USA
β-Catenin	1:1000	CST
Vimentin	1:1000	CST
p-EGFR	1:1000	CST
EGFR	1:1000	CST
SOS-1	1:1000	CST
ERK1/2	1:1000	CST
p-ERK1/2	1:1000	CST
β-actin	1:5000	Abcam
Tubulin	1:10000	Abcam

### Tumor xenograft mouse model

All experiments were approved by the Shihezi University Animal Ethics Committee and were performed in accordance with the US guidelines. Male C57BL/6 mice (6 weeks old) were obtained from the Shanghai Animal Laboratory Center (Shanghai, China). U87 cells (0.2 mL; 2×10 ^7^ cells) were injected subcutaneously into the left armpit of each mouse. Treatments were initiated when tumors reached approximately 90 mm ^3^, and the mice were randomly divided into two groups according to treatment: (1) control group (saline, once a day, intraperitoneally) and (2) β-sitosterol group (80 mg/kg, once a day, intraperitoneally). The tumor volume was calculated as follows: tumor volume (mm ^3^)=length×(width ^2^)/2. After 28 days, the mice were sacrificed, and the tumors were removed, weighed, photographed and prepared for paraffin embedding.

### Hematoxylin-eosin (HE) staining

After paraffin embedding the tumor tissue, tissue sections with a thickness of 5 μm were prepared. The sections were dewaxed by xylene and hydrated with ethanol, stained with hematoxylin for 5 min, rinsed and placed in 1% hydrochloric acid ethanol for 15 s to achieve differentiation. After washing with distilled water, the sections were stained with eosin for 2 min and rinsed with distilled water again. The slices were dehydrated with gradient ethanol, soaked with xylene and sealed with neutral gum. A light microscope (Olympus) was used to take images.

### Statistical analysis

All statistical analyses were performed by using GraphPad Prism 8 software (GraphPad Software, La Jolla, USA). Data and results are expressed as the mean±SD of five samples. Statistically significant differences were calculated using Student’s *t* test or one-way ANOVA. A *P* value less than 0.05 was considered statistically significant.

## Results

### β-Sitosterol inhibited proliferation in glioma cells

**Figure 1:**
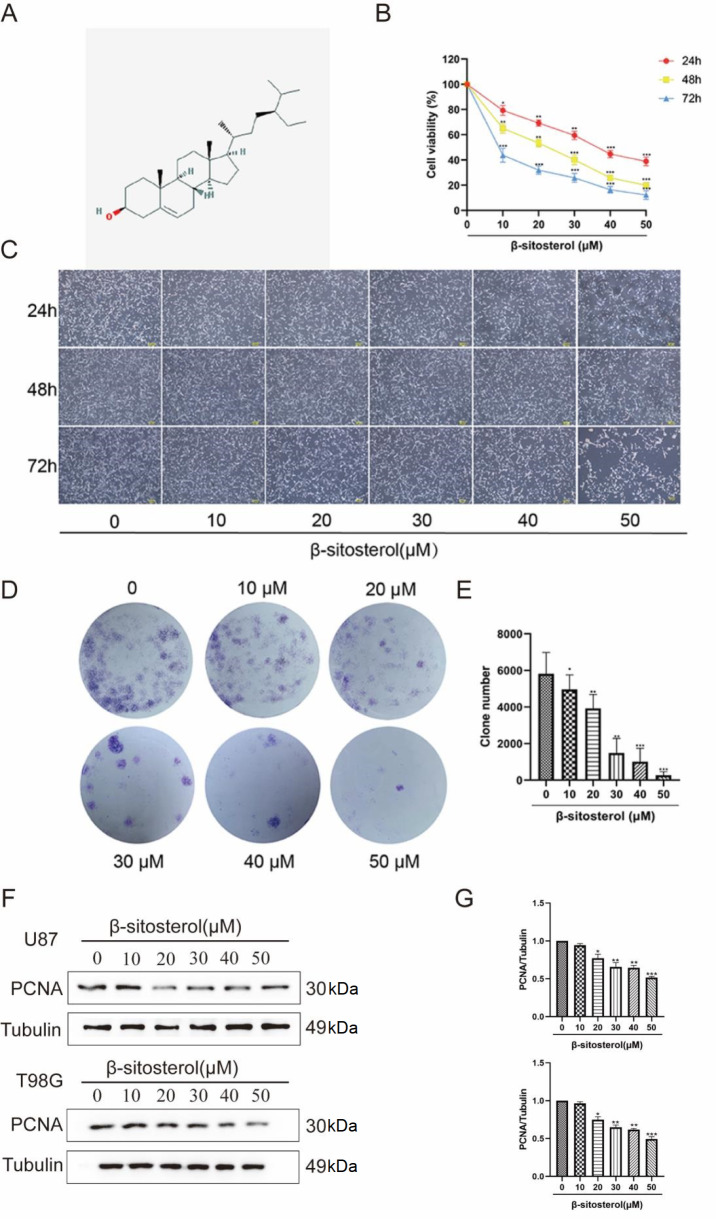
Figure 1 β-Sitosterol effectively inhibited the proliferation of the glioma cell line glioma (A) Chemical structure of β-sitosterol. (B) U87 cells were treated with β-sitosterol at different concentrations (0, 10, 20, 30, 40 and 50 μM) for 24, 48 and 72 h, and cell viability was detected by the MTT assay. The negative control group was treated with an equal volume of medium containing anhydrous ethanol. (C) U87 cells were treated with β-sitosterol at different concentrations for 24, 48 and 72 h, and the morphological changes in the cells were observed under a light microscope. (D,E) After U87 cells were treated with different concentrations of β-sitosterol for 48 h, changes in cell proliferation and colony formation ability in vitro were observed by plate cloning experiments and the corresponding statistical graph. All data are expressed as the mean±SD (n=5; *P<0.05, **P<0.01, ***P<0.001). (F,G) After treating U87/T98G cells with different concentrations of β-sitosterol for 48 h, the expression level of proliferation-associated protein PCNA was detected by western blot analysis, and the corresponding statistical graphs were drawn. All data were expressed as the mean±SD (n=5; *P<0.05, **P<0.01, ***P<0.001).

The chemical structure of β-sitosterol is shown in Figure 1A. To investigate the effects of β-sitosterol on glioma *in vitro*, U87 cells were treated with different concentrations of β-sitosterol (0, 10, 20, 30, 40, and 50 μM) for 24, 48 and 72 h. Then, the viabilities of the treated cells were detected by the MTT assay. The results showed a significant concentration-dependent and time-dependent reduction in cell viability ( Figure 1B). The IC50 values calculated from the MTT assay data of U87 cells were 35.82 μM at 24 h, 31.75 μM at 48 h, and 9.43 μM at 72 h. To confirm the inhibitory effect of sitosterol, the cell morphology was observed under a microscope after drug treatment. Compared with that in the control group, the number of U87 cells in the β-sitosterol-treated group was significantly reduced. As the drug concentration increased, cells showed irregular cell outlines and cell shrinkage, and some even fell off the surface of the culture dish ( Figure 1C). Next, a colony formation assay was performed to examine the effect of β-sitosterol on proliferation. β-Sitosterol significantly reduced the colony number of U87 cells compared with the control ( Figure 1D,E). Finally, we also detected the level of pro-proliferative PCNA by western blot analysis. The results showed that β-sitosterol significantly inhibited the level of PCNA, depending on the concentration of β-Sitosterol ( Figure 1F,G). These results demonstrate that β-sitosterol effectively inhibited glioma cell proliferation.

### β-Sitosterol induced apoptosis in glioma cells

**Figure 2:**
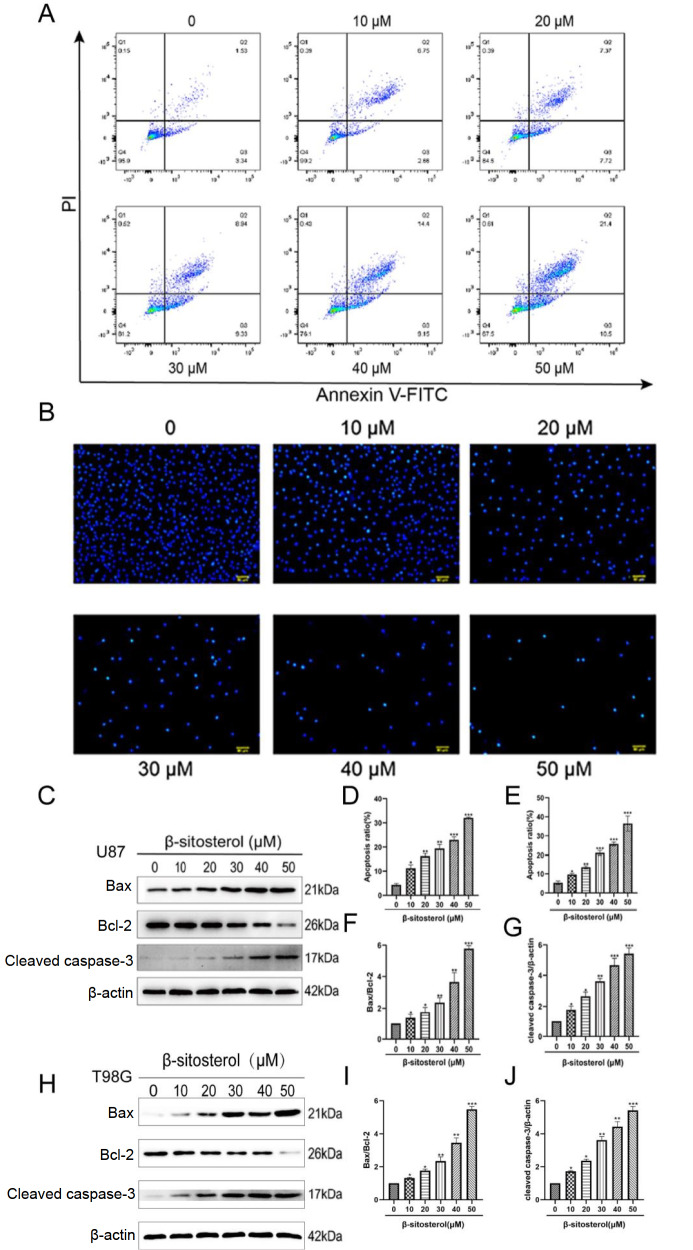
Figure 2 β-Sitosterol significantly increased the apoptosis level of the glioma cell line (A,D) The apoptosis rate of U87 cells was measured by flow cytometry 48 h after β-sitosterol intervention, and the changes were statistically analysed. (B,E) After U87 cells were treated with β-sitosterol at different concentrations for 48 h, Hoechst 33342 staining was observed under a fluorescence microscope to observe the changes in the cell apoptosis rate. Statistical charts were drawn based on the data. (C,F–J) Forty-eight hours after U87/T98G cells were treated with different concentrations of β-sitosterol, the protein expression levels of Bax, Bcl-2 and cleaved caspase-3 were detected by western blot analysis. The bar charts show the changes in Bax/Bcl-2 protein expression and cleaved caspase-3 protein expression. All data are expressed as the mean±SD (n=5; *P<0.05, **P<0.01, ***P<0.001).

To determine whether apoptosis was involved in the antiproliferative effect of β-sitosterol, a Hoechst 33342 staining assay was performed. Figure 2B,E indicated that typical apoptosis morphology, such as shrunken cells, nuclear condensation and fragmentation, and improved brightness, was observed in the β-sitosterol groups. To quantify the apoptosis triggered by β-sitosterol, cells were evaluated by the annexin V-FITC/PI double-staining assay. As shown in Figure 2A,D, the apoptotic rates were markedly increased in a dose-dependent manner compared to those in the untreated groups, and the rate of apoptosis was observed to range from 3.32% (control group) to 25.54%. The results showed that β-sitosterol dose-dependently upregulated the hallmark proteins of apoptosis, including Bax and cleaved caspase-3, and significantly suppressed Bcl-2 levels depending on the concentration of β-sitosterol ( Figure 2C,F–J).

### β-Sitosterol induced G2/M cell cycle arrest in glioma cells

**Figure 3:**
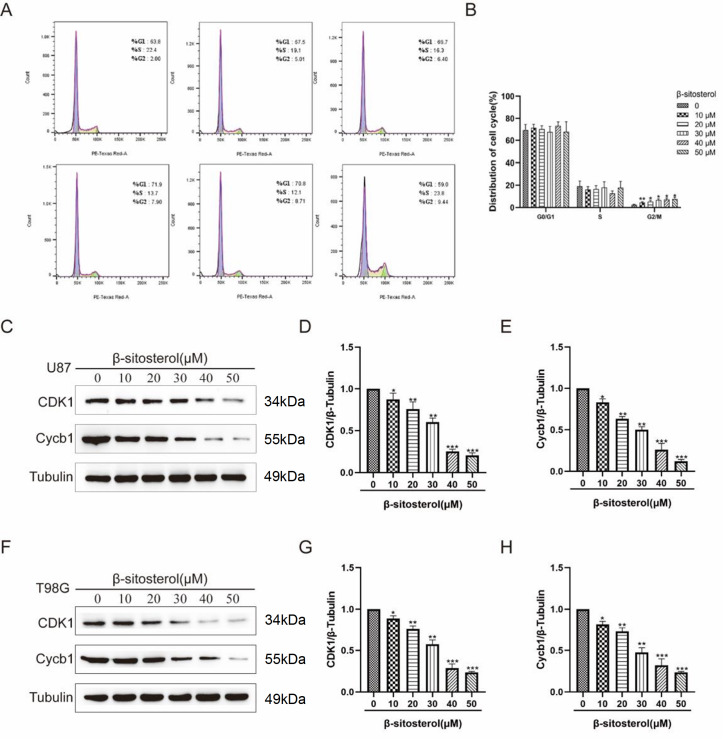
Figure 3 Effect of β-sitosterol on the cycle progression of the glioma cell line glioma (A,B) U87 cells were treated with β-sitosterol at different concentrations for 48 h, and G2/M phase arrest was detected by flow cytometry. (C‒H) Forty-eight hours after U87/T98G cells were treated with different concentrations of β-sitosterol, the protein expression levels of CDK1 and Cycb1 were detected by western blot analysis. The bar charts show the changes in CDK1/Tubulin protein expression and Cycb1/Tubulin protein expression. All data are expressed as the mean±SD (n=5; *P<0.05, **P<0.01, ***P<0.001).

To further investigate the inhibitory effects of β-sitosterol on the proliferation of glioma cells, we analyzed the cell cycle distribution by flow cytometry after 48 h of treatment with β-sitosterol. The data showed that the percentages of cells in the G2/M phase in the treatment group were significantly higher than those in the control group ( Figure 3A,B). Western blot results revealed that following β-sitosterol treatment, the levels of cyclin B1 and CDK1 were reduced, and the effect was concentration dependent ( Figure 3C–H), indicating that β-sitosterol induced G2/M phase arrest in glioma cells.

### β-Sitosterol inhibited the cell migration of glioma cells

**Figure 4:**
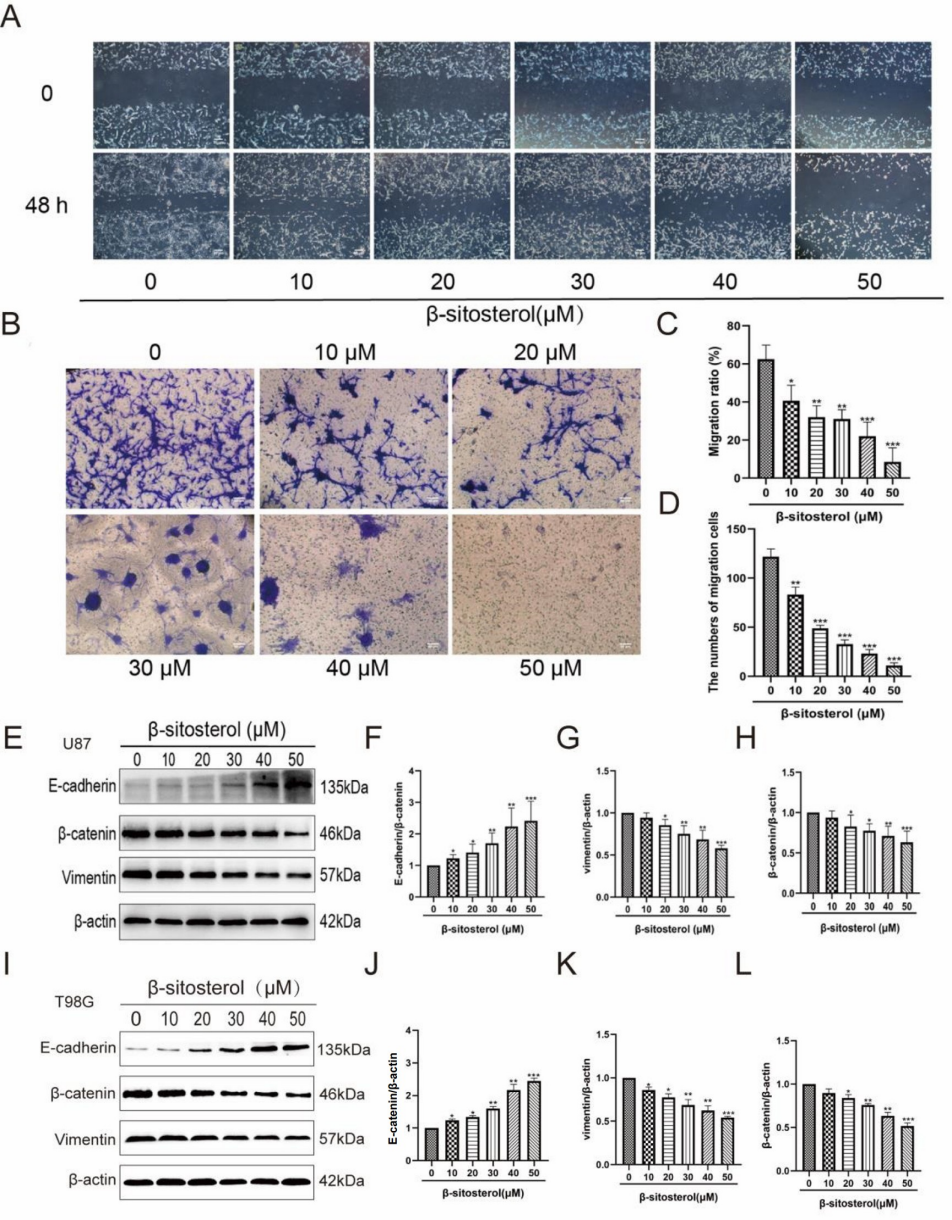
Figure 4 β-Sitosterol significantly inhibited the migration of glioma and blocked the progression of EMT (A,C) Scratch test: the change in the scratch area of cells treated with β-sitosterol was recorded under a light microscope and statistically analysed. (B,D) Transwell experiments were performed by recording the number of cell permeable membranes and recording the change in the migration ability of cells after 48 h of β-sitosterol intervention. (E–L) Forty-eight hours after U87 cells were treated with different concentrations of β-sitosterol, the protein expression levels of E-cadherin, β-catenin and vimentin were detected by western blot analysis. The histogram shows the expression changes of E-cadherin, β-catenin and vimentin in U87/T98G cells after 48 h of β-sitosterol intervention at various concentrations. All data are expressed as the mean±SD (n=5; *P<0.05, **P<0.01, ***P<0.001).

To identify the effect of β-sitosterol on the cellular migration activity of glioma cells, cells were treated with different concentrations of β-sitosterol for 48 h. The wound healing assay was applied to measure the effect of β-sitosterol on the migration of U87 cells. The results showed that β-sitosterol significantly decreased U87 cell migration and decreased the percentage of the scratched area compared with the control group ( Figure 4A,C). The migratory ability of β-sitosterol was further confirmed by the Transwell assay ( Figure 4B,D). We noticed an evident dose-dependent inhibitory effect of β-sitosterol on the decrease in migration activities of U87 cells. EMT is increasingly being regarded as a mechanism by which tumor cells can reactivate EMT programs, which increases their aggressiveness [29]. To identify the effect of β-sitosterol on EMT, U87 cells were treated with different concentrations of β-sitosterol for 48 h. We attempted to confirm the expressions of specific EMT markers through western blot analysis. The results revealed that β-sitosterol treatment significantly increased the expression of E-cadherin and reduced the expressions of vimentin and β-catenin in a dose-dependent manner.

### Network pharmacology predicted potential signaling pathways in the inhibitory effect of β-sitosterol on glioma

**Figure 5:**
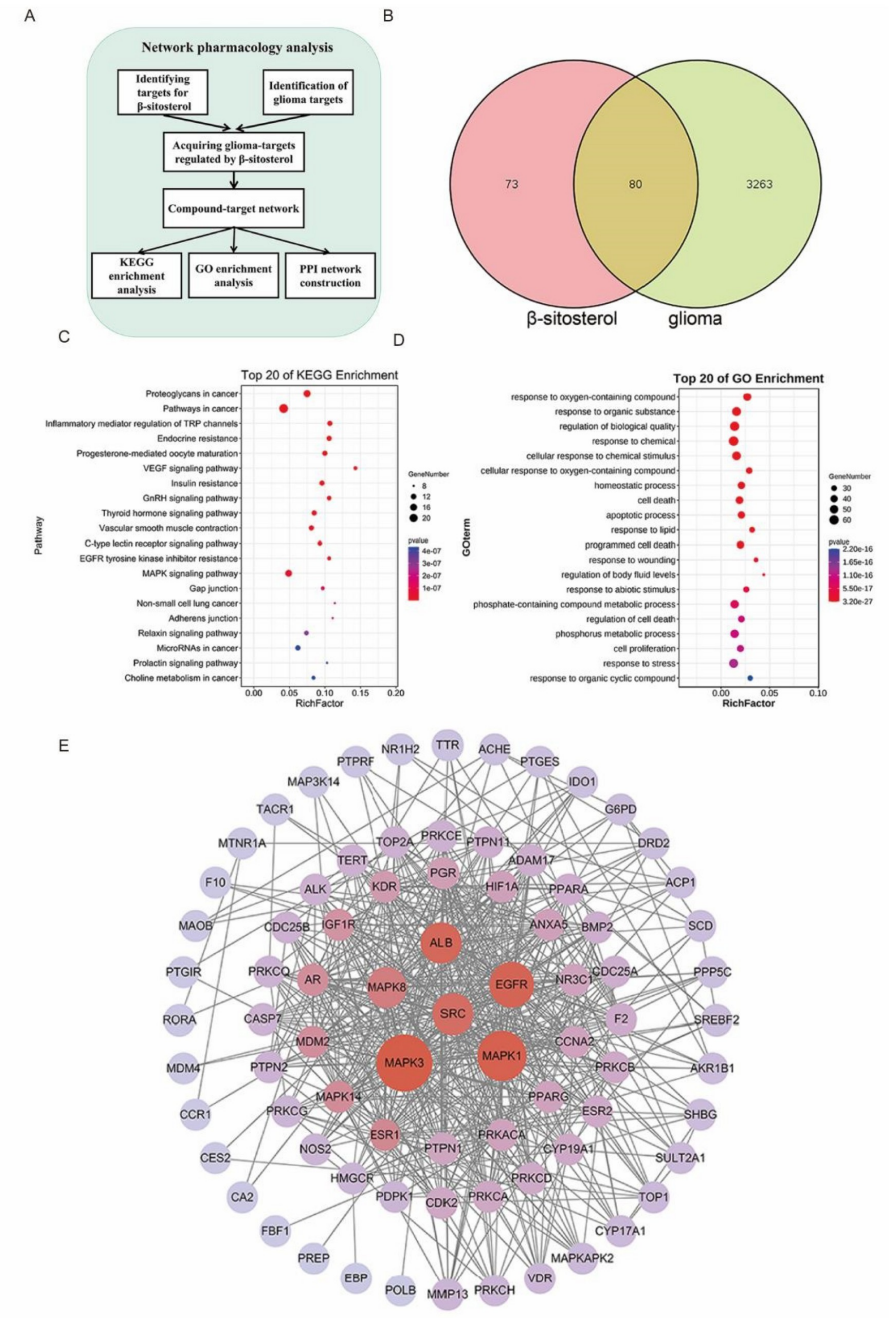
Figure 5 Network pharmacology and biological function analysis of β-sitosterol in glioma (A) Description of the network pharmacology technology and process involved in this experiment. (B) The Venn diagram shows that β-sitosterol and glioma have a total of 80 common targets. (C,D) The enrichment results of the first 20 KEGG pathways and the biological functions of GO were predicted through network pharmacological screening. (E) The results of protein-protein interactions predicted by network pharmacology were ranked according to P values.

Our results confirmed that β-sitosterol has an inhibitory effect on glioma cells. However, the potential mechanisms are still unclear. To further understand the potential molecular mechanism, we focused on network pharmacology, which is an effective tool for studying the multiple interactions between compounds and diseases. The protocol of network pharmacology analysis is shown in Figure 5A. A total of 153 potential protein targets of β-sitosterol were predicted using an online database. Glioma targets were collected from the DisGeNET, OMIM, and GeneCards databases, and 3343 known glioma targets were collected after removing duplicates. Among these targets, 80 overlapping targets between β-sitosterol and glioma were selected as β-sitosterol-regulated glioma targets ( Figure 5B).

To further illustrate the main biological functions of the core targets, GO and KEGG functional enrichment analyses were performed. According to the enrichment factor and *P* value<0.05, the top 20 pathways were selected as the main pathways. It was found that these targets were closely related to cancer processes, such as proteoglycans in cancer, pathways in cancer, VEGF signaling pathway, EGFR tyrosine kinase inhibitor resistance, and MAPK signaling pathway, by KEGG enrichment analysis ( Figure 5C). GO enrichment analysis ( Figure 5D) showed that these targets were mainly enriched in cellular response to oxygen-containing compounds, regulation of biological quality, homeostatic process, apoptotic process and so on. The PPI network of the 80 potential targets was established through the STRING database and was visualized by Cytoscape 3.6.1. MAPK3, MAPK1, and EGFR were ranked among the top 3 target proteins ( Figure 5E), and were also the key proteins tightly associated with cancer.

### Transcriptomics analysis revealed the signaling pathways involved in the inhibitory effect of β-sitosterol on glioma

**Figure 6:**
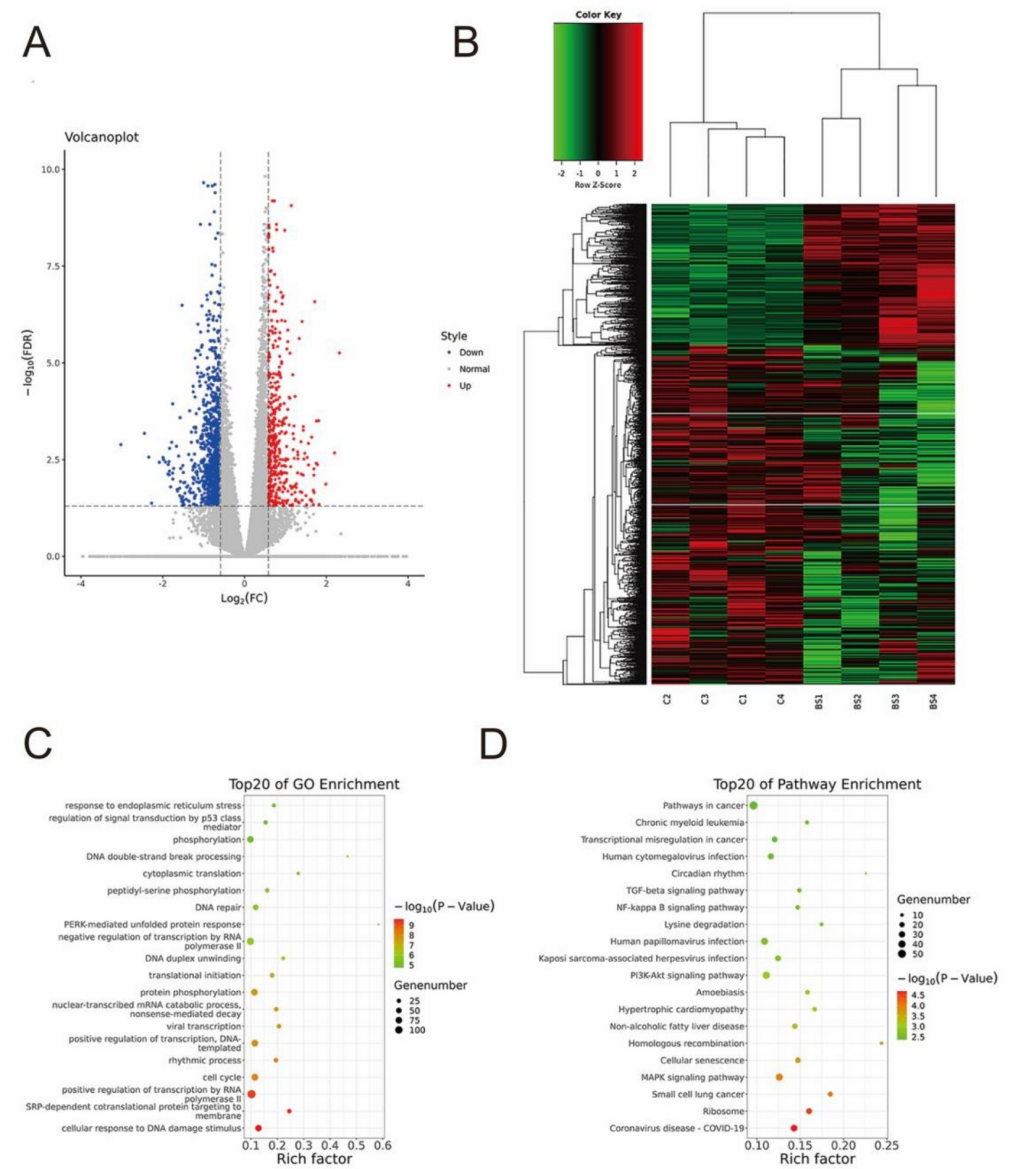
Figure 6 Transcriptomics analysis revealed the signaling pathways involved in the inhibitory effect of β-sitosterol on glioma (A,B) Tran-scriptomics predicted the volcano map and Heatmap results of gene changes in U87 cell lines treated with β-sitosterol for 48 h. (C,D) After β-sitosterol intervention in U87 cell lines for 48 h, the transcriptome was used to predict the top 20 KEGG enrichment pathways and GO biological functions.

Due to the limitations of network pharmacology, we also used transcriptomics methods to compensate for the above shortcomings and further clarify the potential mechanism of β-sitosterol in glioma. The cut-off criteria were set as *P*<0.05 and |log2FC| > 0.585 in this study to screen out DEGs. In our study, a total of 1468 DEGs were identified in the treatment group versus the control group, 429 of which were upregulated and 1029 of which were downregulated. Therefore, these 1468 DEGs were considered therapeutic targets of glioma. As shown in Figure 6A, the blue dots correspond to significantly downregulated DEGs, the red dots correspond to significantly upregulated DEGs, and the gray dots indicate DEGs that were not statistically significant. Figure 6B displays the heatmap distribution of these DEGs after treatment with β-sitosterol. Green indicates low expression of DEGs, whereas red indicates high expression of DEGs. Transcriptomics-based enrichment analysis of DEGs was further performed to reveal the potential mechanisms involved in cancer initiation and progression.

The top 20 GO enriched pathways revealed that the potential targets of β-sitosterol were involved in phosphorylation, DNA double-strand break processing, cytoplasmic translation, peptidyl-serine phosphorylation, DNA repair, and others ( Figure 6C). The results of KEGG pathway enrichment indicated that the therapeutic targets of β-sitosterol were mainly enriched in the MAPK signaling pathway, PI3K-Akt signaling pathway, NF-kappa B signaling pathway, TGF-beta signaling pathway and so on ( Figure 6D). Collectively, these data highlight the role of the MAPK signaling pathway in the regulation of glioma by β-sitosterol. SOS1 is a key molecule of the MAPK signaling pathway and can highly regulate the MAPK signaling pathway. The results showed that SOS1 expression was downregulated and significantly enriched in the MAPK signaling pathway.

### Molecular docking verified the effect of β-sitosterol on key targets

**Figure 7:**
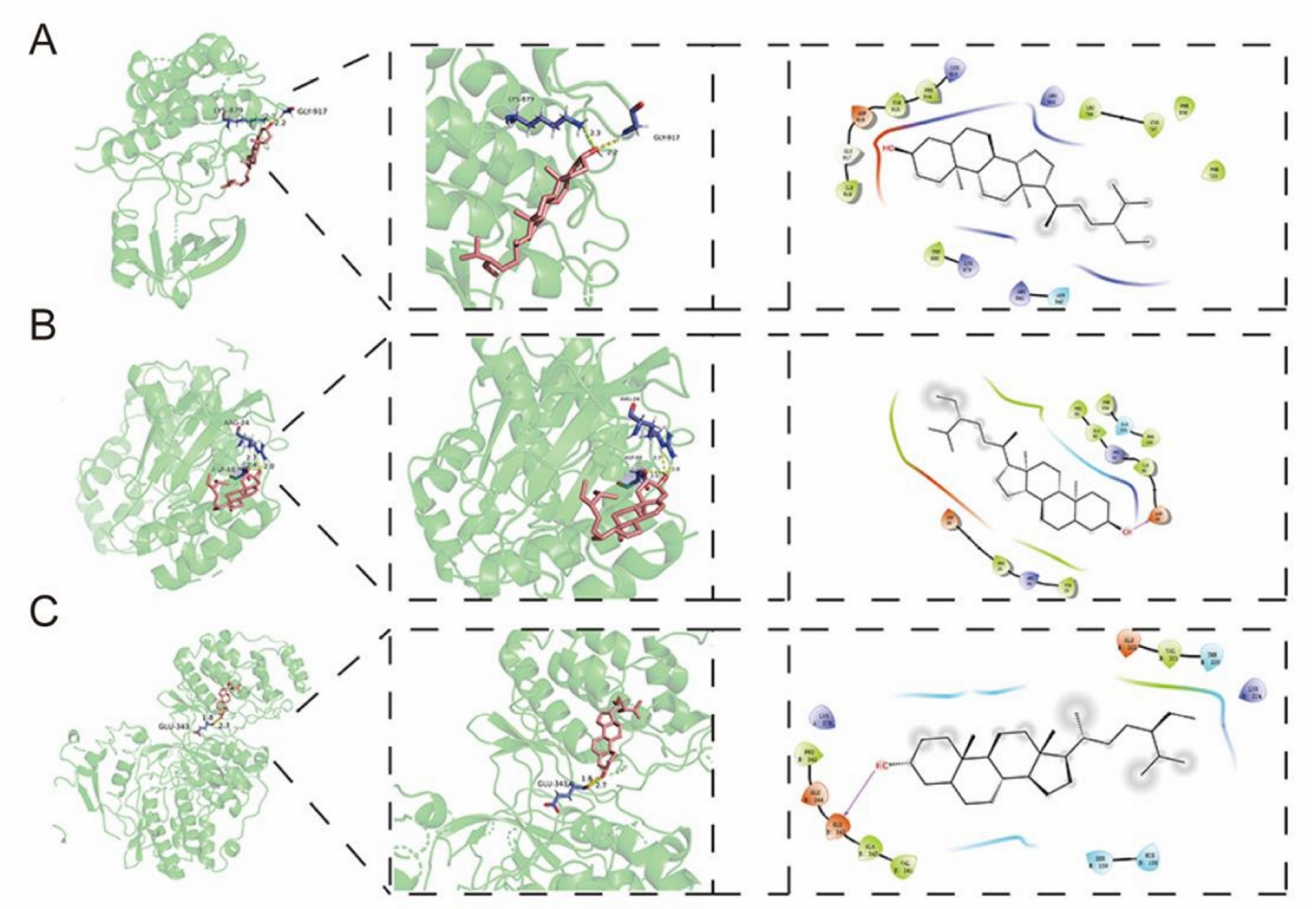
Figure 7 Two-dimensional and three-dimensional docking models of EGFR, MAPK1 and MAPK3 with β-sitosterol and ligands

Molecular docking was used to analyze the interaction between β-sitosterol and the core targets (EGFR, MAPK1 and MAPK3). The docking results showed that β-sitosterol had good binding ability to the targets. As shown in Figure 7A, there were 2 amino acid residues (LYS879 and GLY917) at the EGFR active site that interacted with β-sitosterol to form hydrogen bonds. Meanwhile, hydrogen bonds were formed between β-sitosterol and 2 amino acid residues at the MAPK1 active site, including ARG-24 and ASP-88 ( Figure 7B). Furthermore, β-sitosterol maintained its favorable binding with MAPK3 by forming hydrogen bonds with the residues GLU-343 ( Figure 7C).

### β-Sitosterol downregulated the expression of the EGFR/MAPK signaling pathway in glioma cells

**Figure 8:**
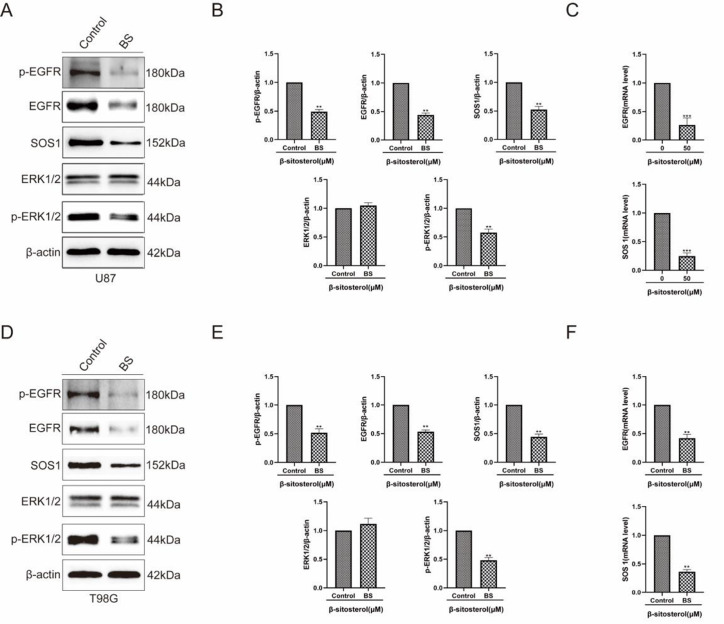
Figure 8 β-Sitosterol acted on glioma cells by downregulating the EGFR/MAPK signaling pathway (A,D) Western blot analysis was used to detect the protein expressions of specific targets (EGFR, p-EGFR, SOS1, ERK1/2 and P-ERK1/2) in the EGFR/MAPK pathway after 48 h of β-sitosterol intervention in U87 cells. (B,E) Quantitative statistical results of expression changes of target proteins and pathway characteristic proteins. (C,F) Quantitative statistical results of expression changes in mRNA. All data are expressed as the mean±SD (n=5; *P<0.05, **P<0.01).

Combined with the above results, the EGFR/MAPK signaling pathway was believed to be responsible for β-sitosterol-mediated glioma cell inhibition. Thus, we verified whether β-sitosterol treatment affected the EGFR/MAPK signaling pathway by western blot analysis and RT-PCR analysis. As shown in Figure 8,p-EGFR, EGFR, SOS1, ERK1/ERK2 and p-ERK1/ERK2 were dramatically downregulated, while β-sitosterol had no effect on the expression levels of total ERK1/ERK2 after treatment. In addition, we tested whether β-sitosterol affects the transcription of the *EGFR* and *SOS1* genes. The results showed that β-sitosterol reduced the mRNA levels of *EGFR* and *SOS1* ( Figure 8C). Taken together, these results indicated that β-sitosterol significantly downregulated the EGFR/MAPK pathway in glioma cells.

### β-Sitosterol suppressed tumor growth in a xenograft tumor model

**Figure 9:**
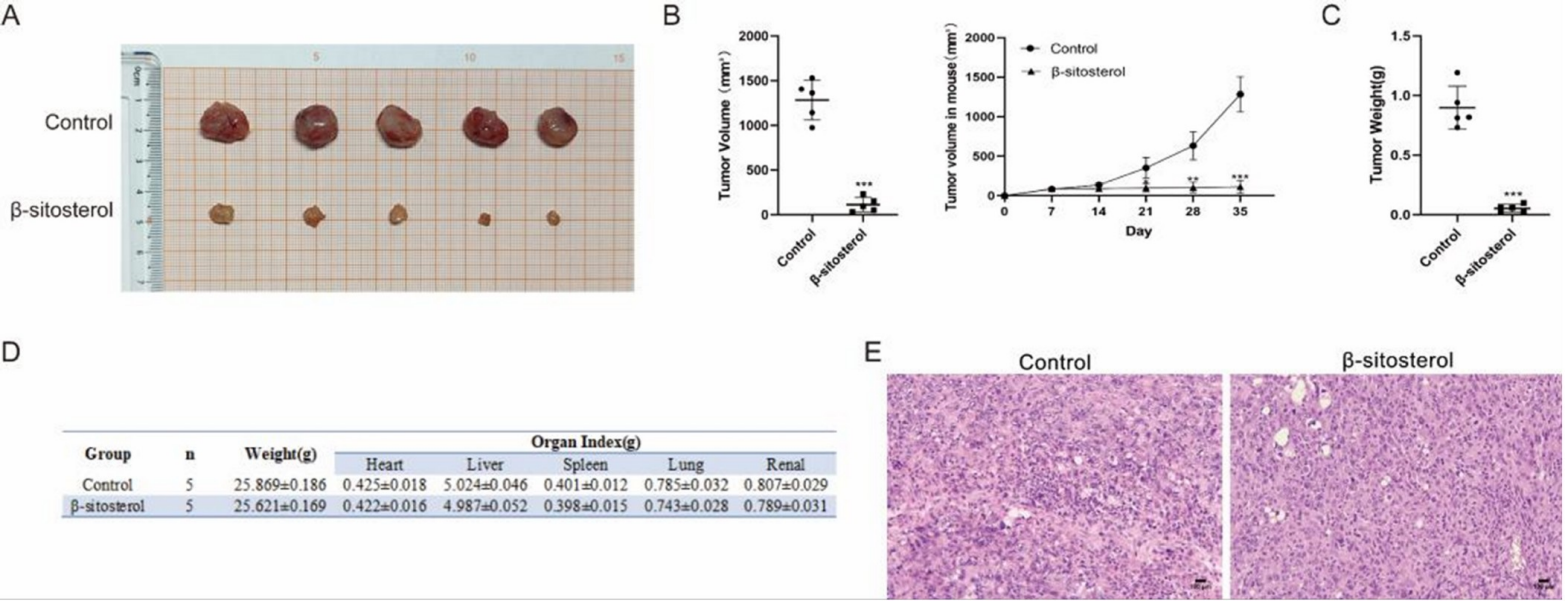
Figure 9 β-Sitosterol inhibited tumor growth in a xenograft model (A) Photograph of tumors in the control and β-sitosterol-treated groups. (B) Mean tumor size and growth curve of the control and β-sitosterol-treated groups. (C) Mean tumor weight of the control and β-sitosterol-treated groups. (D) Vital organ index of the control and β-sitosterol-treated groups. (E) HE staining of the control and β-sitosterol-treated groups. All data are expressed as the mean±SD (n=5; ***P<0.001).

To further confirm the inhibitory effect of β-sitosterol on glioma, we injected U87 cells into C57BL/6 mice to establish a subcutaneous tumor model. The results showed that the average tumor size was 1282.164±221.869 mm ^3^ in the control group and 113.126±81.405 mm ^3^ in the β-sitosterol group ( Figure 9A). The mean tumor weight was 0.899±0.180 g in the control group and 0.054±0.033 g in the β-sitosterol-treated group. Tumor volume was significantly decreased after drug therapy ( Figure 9B). The mean tumor weight of the β-sitosterol treatment group was significantly lower than that of the control group ( Figure 9C). At the same time, there was no significant difference in body weight between the β-sitosterol treatment group and the control group, and the organ index was basically similar to that of the control group, indicating that the treatment had no significant toxicity to animals ( Figure 9D). HE staining was performed on the tumor tissues of the control group and β-sitosterol treatment group. The results showed that the control group had disordered tissue structure, the continuity of the arrangement was destroyed to varying degrees, the cell volume was increased, the size was different, the nuclei were divided more, the nucleoli were increased, the nucleo-plasma ratio was increased, and the background was multiple vacuoles and necrosis. In contrast, the β-sitosterol treatment group showed the characteristics of cell structure, intact cell continuity, uniform cell distribution and clear structure ( Figure 9E). This is consistent with the trend of *in vitro* experiments.

## Discussion

GBM is the most aggressive brain tumor with a very poor prognosis [30]. The 5-year survival rate is only 5% [31]. Therefore, there is an urgent need to identify the potential biomarkers and molecular mechanisms of glioma. For a long time, Chinese herbal medicines have been recognized for their minimal side effects, multiple targets, and multiple pathways, and they are an important source of chemical entities supporting drug discovery [32]. β-Sitosterol, a phytosterol that has been proven to be beneficial, inhibits the migration and invasion of human multiple myeloma, pancreatic cancer and prostate cancer by downregulating the expression of VEGF and CDH1 [33]. However, the effect of β-sitosterol on glioma and its underlying molecular mechanism are still unclear.

Tumors are diverse and heterogeneous, but they all have the ability to proliferate beyond the limits of normal tissue growth. Abnormal regulation of a limited number of key pathways that control cell proliferation and survival is a necessary condition for the establishment of all tumors [34]. Cell cycle dysregulation is the main cause of malignant proliferation of glioma cells and is a classic therapeutic target for tumor drug development [35]. Cyclin B1 protein level gradually increases at G2, which is the activator of G2/M checkpoint, forming an active cyclin B1-CDK1 complex into mitosis [36]. Anticancer agents can result in cell cycle arrest at the G2/M phase through TP53 dependent and independent mechanisms to block the cells’ entry into mitosis [37]. Drug-induced apoptosis of cancer cells is mainly mediated by mitochondrial (intrinsic) or death receptor (extrinsic) mechanisms via caspase activation [38]. Mitochondrial-mediated caspase cascade activation is an important apoptosis pathway [39]. When cells are initiated by internal apoptotic stimulating factors, Bcl-2 family proteins regulate membrane potential to control the permeability of the outer mitochondrial membrane [40]. Subsequently, cytochrome C from the mitochondria is released into the cytoplasm to activate Apaf-1, caspase-9, and caspase-3, leading to cell apoptosis [41]. Our results revealed that β-sitosterol promoted apoptosis through the mitochondria-mediated apoptotic signaling pathway. At the same time, β-sitosterol induced cell cycle arrest at the G2/M phase in U87 cells, which may contribute to growth inhibition. Cancer cells that have undergone EMT are more aggressive, showing higher invasiveness, stem cell-like characteristics, and resistance to apoptosis [42]. Activation of EMT can promote the development of a phenotype towards increased invasiveness by changing the microenvironment and inhibit the sensitivity of tumour cells to chemotherapy, in which mesenchymal cells exhibit higher resistance to treatment regimens such as chemotherapy and immunotherapy. During transformation, the expression or function of epithelial genes such as E-cadherin-specific cytokeratin and occlusion-1 (ZO-1) was lost, while the expressions of genes that define the mesenchymal phenotype (such as vimentin, fibronectin, N-cadherin β1 and β3 integrin) are increased [43]. β-Sitosterol promoted E-cadherin expression and inhibited β-catenin and vimentin expressions, suggesting that β-sitosterol was involved in the EMT process and its role in the development and metastasis of glioma.

In recent years, the emergence and continuous development of systems biology has provided a new way of thinking for drug discovery-multi-target drugs. At the same time, the understanding and research of network pharmacology also revealed that multi-target drugs are more effective and will continue to evolve in the contemporary model of drug discovery. Multi-target drug therapy means that drugs act on multiple targets in the disease network at the same time, and produce synergistic effects on each target, so that the total effect is greater than the sum of each single effect, and the best therapeutic effect is achieved [44]. Therefore, drug development should be tuned to a multi-target model with synergistic effects, rather than blocking or activating a single target. The treatment of tumors with multi-target kinase inhibitors illustrates this concept [45]. β-Sitosterol is a phytosterol that plays an important role in the prevention and treatment of cancer. We explored the potential molecular mechanisms of β-sitosterol in glioma through network pharmacology and transcriptomics. MAPK3, MAPK1 and EGFR were the top 3 target proteins predicted by network pharmacology. Furthermore, molecular docking was used to verify the interaction between β-sitosterol and key targets, and the results showed that β-sitosterol formed a stable complex with key targets. Transcriptomics results showed that *SOS1* mRNA expression was downregulated and significantly enriched in the MAPK signaling pathway. SOS1 is a key downstream molecule of EGFR that highly regulates the MAPK signaling pathway. Combined with the above research results, the EGFR/MAPK signaling pathway was believed to be responsible for β-sitosterol-mediated glioma inhibition. β-Sitosterol can exert synergistic effects by inhibiting multiple targets. Many studies have confirmed that the EGFR/MAPK signaling pathway plays crucial roles in the regulation of cancer. Inhibiting the EGFR/MAPK pathway could reduce the expression of cyclin D1, thereby inhibiting the proliferation of NSCLC and the G1/S transition [46]. EGFR overexpression led to the dimerization and autophosphorylation of EGFR, which blocked the phosphorylation of EGFR and its downstream signaling: the phosphorylation of MEK and ERK proteins. Importantly, inhibiting MAPK signaling led to an imbalance in Bcl-2/Bax, which led to a cascade of caspase pathways [47]. In addition, LINC01225 increased the protein level of EGFR by binding to EGFR, thereby activating the EGFR/MAPK signaling pathway and promoting the occurrence and metastasis of hepatocellular carcinoma [48]. RNF128 regulates the expression of MMP-2 by activating the EGFR/MAPK signaling pathway, thereby promoting the invasion and metastasis of esophageal squamous cell carcinoma [49]. The study found that β-sitosterol treatment significantly inhibited the EGFR/MAPK signaling pathway, indicating that the EGFR/MAPK signaling pathway was involved in β-sitosterol-mediated proliferation, cell cycle arrest, apoptosis and migration in U87 cells. Furthermore, the apparent antitumour effect of β-sitosterol was observed *in vivo* in the U87 xenograft nude mouse model in our study. Collectively, our findings reveal β-sitosterol as a promising therapeutic option for glioma.

**Figure 10:**
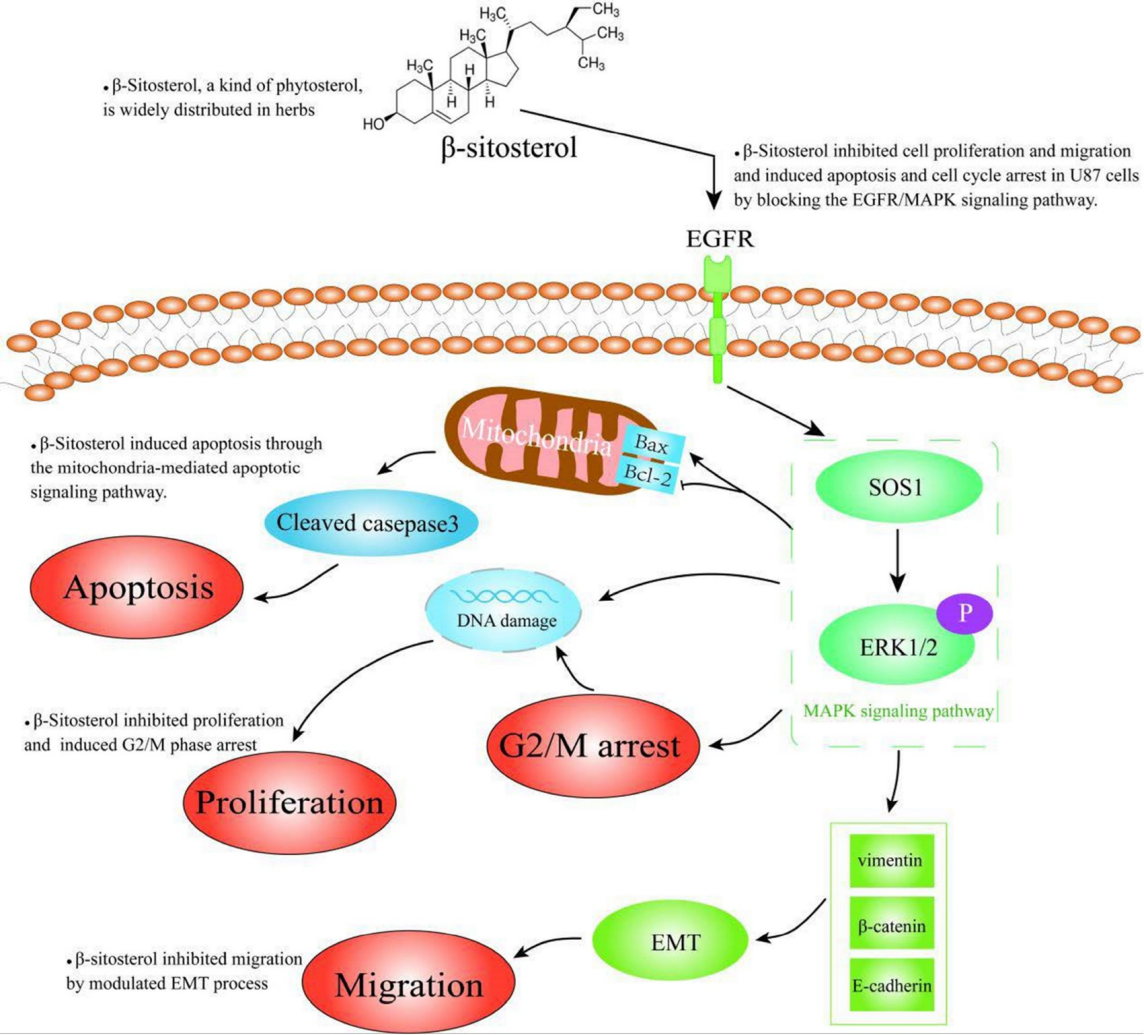
Figure 10 β-Sitosterol inhibited cell proliferation and migration and induced apoptosis and cell cycle arrest by blocking the EGFR/MAPK signaling pathway in glioma In glioma, β-sitosterol inhibits the oxidation of fatty acids through cell cycle arrest by blocking the expression of EGFR/MAPK signaling pathway in glioma, leading to intracellular lipid accumulation, thereby inhibiting cell proliferation, blocking the G2/M cell cycle process and the expression of related cyclins, thereby inhibiting cell proliferation and migration. Induce cell apoptosis and cell cycle arrest.

In summary, in this study we reported for the first time to our knowledge that β-sitosterol inhibited U87 cell proliferation and migration and induced apoptosis and cell cycle arrest in U87 cells by blocking the EGFR/MAPK signaling pathway ( Figure 10). β-Sitosterol is a potential therapeutic agent for the management of glioma.

## References

[1] Chen W, Lei C, Liu P, Liu Y, Guo X, Kong Z, Wang Y (2020). Progress and prospects of recurrent glioma: a recent scientometric analysis of the web of science in 2019. World Neurosurg.

[2] Grauwet K, Chiocca EA (2016). Glioma and microglia, a double entendre. Nat Immunol.

[3] Hanif F, Muzaffar K, Perveen K, Malhi SM, Simjee ShU. Glioblastoma multiforme: a review of its epidemiology and pathogenesis through clinical presentation and treatment.
Asian Pac J Cancer Prev. 2017, 18: 3–9. https://doi.org/10.22034/APJCP.2017.18.1.3.

[4] Sattiraju A, Sai KKS, Mintz A. Glioblastoma stem cells and their microenvironment.
Adv Exp Med Biol, 2017, 1041, 119–140. https://www.doc88.com/p-0744870526908.html.

[5] Reitman Z, Winkler F, Elia A (2018). New directions in the treatment of glioblastoma. Semin Neurol.

[6] Xiao Y, Liu Y, Lai Z, Huang J, Li C, Zhang Y, Gong X (2021). An integrated network pharmacology and transcriptomic method to explore the mechanism of the total rhizoma coptidis alkaloids in improving diabetic nephropathy. J EthnoPharmacol.

[7] Chikara S, Nagaprashantha LD, Singhal J, Horne D, Awasthi S, Singhal SS (2018). Oxidative stress and dietary phytochemicals: role in cancer chemoprevention and treatment. Cancer Lett.

[8] Vo TK, Ta QTH, Chu QT, Nguyen TT, Vo VG (2020). Anti-hepatocellular-cancer activity exerted by β-sitosterol and β-sitosterol-glucoside from
*Indigofera zollingeriana* Miq. Molecules.

[9] Agarwal G, Carcache PJB, Addo EM, Kinghorn AD (2020). Current status and contemporary approaches to the discovery of antitumor agents from higher plants. Biotechnol Adv.

[10] Iqbal J, Abbasi BA, Ahmad R, Mahmood T, Kanwal S, Ali B, Khalil AT (2018). Ursolic acid a promising candidate in the therapeutics of breast cancer: current status and future implications. Biomed Pharmacother.

[11] Yin SY, Wei WC, Jian FY, Yang NS (2013). Therapeutic applications of herbal medicines for cancer patients. Evid-Based Complement Alternat Med.

[12] Nguyen NH, Nguyen TT, Ma PC, Ta QTH, Duong TH, Vo VG (2020). Potential antimicrobial and anticancer activities of an ethanol extract from bouea macrophylla. Molecules.

[13] Wright CW, Anderson MM, Allen D, Phillipson JD, Kirby GC, Warhurst DC, Chang HR (1993). Quassinoids exhibit greater selectivity against
*Plasmodium falciparum* than against
*Entamoeba histolytica*,
*Giardia intestinalis* or
*Toxoplasma gondii in vitro*. J Eukaryotic Microbiol.

[14] Ayaz M, Junaid M, Ullah F, Subhan F, Sadiq A, Ali G, Ovais M (2017). Anti-Alzheimer’s studies on β-sitosterol isolated from
*Polygonum hydropiper* L. Front Pharmacol.

[15] Baskar AA, Ignacimuthu S, Paulraj GM, Al Numair KS (2010). Chemopreventive potential of β-sitosterol in experimental colon cancer model: an
*in vitro* and
*in vivo* study. BMC Complement Altern Med.

[16] Cao Z, Wang X, Lu L, Xu J, Li X, Zhang G, Ma Z (2019). β-Sitosterol and gemcitabine exhibit synergistic anti-pancreatic cancer activity by modulating apoptosis and inhibiting epithelial–mesenchymal transition by deactivating Akt/GSK-3β signaling. Front Pharmacol.

[17] Shin EJ, Choi HK, Sung MJ, Park JH, Chung MY, Chung S, Hwang JT (2018). Anti-tumour effects of beta-sitosterol are mediated by AMPK/PTEN/HSP90 axis in AGS human gastric adenocarcinoma cells and xenograft mouse models. Biochem Pharmacol.

[18] Rajavel T, Packiyaraj P, Suryanarayanan V, Singh SK, Ruckmani K, Pandima Devi K (2018). β-Sitosterol targets Trx/Trx1 reductase to induce apoptosis in A549 cells via ROS mediated mitochondrial dysregulation and p53 activation. Sci Rep.

[19] Li S, Zhang B (2013). Traditional Chinese medicine network pharmacology: theory, methodology and application. Chin J Nat Meds.

[20] Zheng J, Wu M, Wang H, Li S, Wang X, Li Y, Wang D (2018). Network pharmacology to unveil the biological basis of health-strengthening herbal medicine in cancer treatment. Cancers.

[21] Vetrivel P, Murugesan R, Bhosale PB, Ha SE, Kim HH, Heo JD, Kim GS (2021). A network pharmacological approach to reveal the pharmacological targets and its associated biological mechanisms of prunetin-5-O-glucoside against gastric cancer. Cancers.

[22] Jiang YH, Jiang LY, Wang YC, Ma DF, Li X (2020). Quercetin attenuates atherosclerosis via modulating oxidized LDL-induced endothelial cellular senescence. Front Pharmacol.

[23] Li L, Dong L, Xiao Z, He W, Zhao J, Pan H, Chu B (2020). Integrated analysis of the proteome and transcriptome in a MCAO mouse model revealed the molecular landscape during stroke progression. J Adv Res.

[24] Huang WC, Su HH, Fang LW, Wu SJ, Liou CJ (2019). Licochalcone an inhibits cellular motility by suppressing e-cadherin and MAPK signaling in breast cancer. Cells.

[25] Wu F, Shao Q, Xia Q, Hu M, Zhao Y, Wang D, Fang K (2021). A bioinformatics and transcriptomics based investigation reveals an inhibitory role of Huanglian-Renshen-Decoction on hepatic glucose production of T2DM mice via PI3K/Akt/FoxO1 signaling pathway. Phytomedicine.

[26] Wang L, Xu H, Liang J, Ding Y, Meng F (2020). An integrated network, RNA sequencing, and experiment pharmacology approach reveals the active component, potential target, and mechanism of gelsemium elegans in the treatment of colorectal cancer. Front Oncol.

[27] Dai Y, Qiang W, Yu X, Cai S, Lin K, Xie L, Lan X (2020). Guizhi fuling decoction inhibiting the PI3K and MAPK pathways in breast cancer cells revealed by HTS2 technology and systems pharmacology. Comput Struct Biotechnol J.

[28] Shen H, Qu Z, Harata-Lee Y, Aung TN, Cui J, Wang W, Kortschak RD (2019). Understanding the mechanistic contribution of herbal extracts in compound kushen injection with transcriptome analysis. Front Oncol.

[29] Aiello NM, Kang Y (2019). Context-dependent EMT programs in cancer metastasis. J Exp Med.

[30] Laug D, Glasgow SM, Deneen B (2018). A glial blueprint for gliomagenesis. Nat Rev Neurosci.

[31] Gao Y, Xuan C, Jin M, An Q, Zhuo B, Chen X, Wang L (2019). Ubiquitin ligase RNF5 serves an important role in the development of human glioma. Oncol Lett.

[32] Li FS, Weng JK (2017). Demystifying traditional herbal medicine with modern approach. Nat Plants.

[33] Bao X, Zhang Y, Zhang H, Xia L (2022). Molecular mechanism of β-sitosterol and its derivatives in tumor progression. Front Oncol.

[34] Evan GI, Vousden KH (2001). Proliferation, cell cycle and apoptosis in cancer. Nature.

[35] Zhang X, Yao Z, Xue Z, Wang S, Liu X, Hu Y, Zhang Y (2022). Resibufogenin targets the ATP1A1 signaling cascade to induce G2/M phase arrest and inhibit invasion in glioma. Front Pharmacol.

[36] Yao J, He C, Zhao W, Hu N, Long D (2021). Circadian clock and cell cycle: cancer and chronotherapy. Acta Histochemica.

[37] Chung TW, Lin SC, Su JH, Chen YK, Lin CC, Chan HL (2017). Sinularin induces DNA damage, G2/M phase arrest, and apoptosis in human hepatocellular carcinoma cells. BMC Complement Altern Med.

[38] Reddy D, Kumavath R, Ghosh P, Barh D (2019). Lanatoside C Induces G2/M cell cycle arrest and suppresses cancer cell growth by attenuating MAPK, Wnt, JAK-STAT, and PI3K/AKT/mTOR signaling pathways. Biomolecules.

[39] Chen Y, Dou C, Yi J, Tang R, Yu T, Zhou L, Luo W (2018). Inhibitory effect of vanillin on RANKL-induced osteoclast formation and function through activating mitochondrial-dependent apoptosis signaling pathway. Life Sci.

[40] Wang YQ, Liu C, Shan ZF, Liu QZ, Cui YS, Yang LQ. Expressions and effects of G250, Bax and Bcl-2 in rats with renal clear cell carcinoma.
Eur Rev Med Pharmacol Sci 2018, 22: 4488–4492. https://doi.org/10.26355/eurrev_201807_15502.

[41] Pan Z, Zhang X, Yu P, Chen X, Lu P, Li M, Liu X (2019). Cinobufagin induces cell cycle arrest at the G2/M phase and promotes apoptosis in malignant melanoma cells. Front Oncol.

[42] Suarez-Carmona M, Lesage J, Cataldo D, Gilles C (2017). EMT and inflammation: inseparable actors of cancer progression. Mol Oncol.

[43] Huang Y, Hong W, Wei X (2022). The molecular mechanisms and therapeutic strategies of EMT in tumor progression and metastasis. J Hematol Oncol.

[44] Jiang Q, Li M, Li H, Chen L (2022). Entrectinib, a new multi-target inhibitor for cancer therapy. Biomed Pharmacother.

[45] Zheng W, Zhao Y, Luo Q, Zhang Y, Wu K, Wang F (2017). Multi-targeted anticancer agents. Curr Top Med Chem.

[46] Fu H, Gao H, Qi X, Zhao L, Wu D, Bai Y, Li H (2018). Aldolase a promotes proliferation and G
_1_/S transition via the EGFR/MAPK pathway in non‐small cell lung cancer. Cancer Commun.

[47] Lu XG, Yang L, Lu CH, Xu ZY, Qiu HF, Wu JJ, Wang JW (2016). Molecular role of EGFR-MAPK pathway in patchouli alcohol-induced apoptosis and cell cycle arrest on A549 cells
*in vitro* and
*in vivo*. Biomed Res Int.

[48] Wang X, Zhang W, Tang J, Huang R, Li J, Xu D, Xie Y (2016). LINC01225 promotes occurrence and metastasis of hepatocellular carcinoma in an epidermal growth factor receptor-dependent pathway. Cell Death Dis.

[49] Gao J, Wang Y, Yang J, Zhang W, Meng K, Sun Y, Li Y (2019). RNF128 promotes invasion and metastasis via the EGFR/MAPK/MMP-2 pathway in esophageal squamous cell carcinoma. Cancers.

